# Developmental and dynamic systems: insights for sex/gender research

**DOI:** 10.1186/s13293-026-00920-x

**Published:** 2026-05-16

**Authors:** Giordana Grossi, Annie Duchesne, Donna L. Maney

**Affiliations:** 1 Department of Psychology, State University of New York at New Paltz, New Paltz, NY USA; 2 Department of Psychology, University of Northern British Columbia, Prince George, British Columbia, Canada; 3 Department of Psychology, University of Quebec in Trois-Rivieres, Trois- Rivieres, QC Canada; 4 Department of Psychology, Emory University, Atlanta, GA USA

**Keywords:** Sex/gender entanglement, Gender/sex, Developmental systems theory, Dynamic systems theory

## Abstract

Recent work incorporating gender into sex differences research has complicated the interpretation of both physiological and behavioral differences between women and men, which are traditionally interpreted as driven by “biological” sex-related variables. Alternative conceptualizations have been proposed to capture the complex relationship between sex and gender, such as sex/gender entanglement. Blurring the boundaries between gender and sex, the organism and its environments, this perspective has created a need for theoretical and methodological approaches that can be used to investigate the emergence of structures and behaviors in complex systems. Here, we review the foundational concepts of developmental and dynamic systems theories, which can serve as frameworks within which to investigate the emergence of complex phenomena. We then provide examples of empirical research programs that demonstrate the benefit of these frameworks in the context of sex/gender research.

## Highlights

1. Recent interest in incorporating gender into research, acknowledging entanglement between gender and sex, necessitates revisiting ways of thinking about how to approach such entanglement.
2. Here, we provide an overview of the foundational concepts of developmental and dynamic systems theories, which can provide frameworks within which to investigate the emergence of complex phenomena.
3. We illustrate how developmental and dynamic systems theories have been applied to the study of sex/gender in both theoretical and empirical contexts.
4. A systems framework can inform the way we design studies, interpret our data, and communicate our findings.

## Introduction

Motivated by the need to acknowledge and understand complex sources of variation in their data, more and more researchers are considering how gender, in addition to sex, may influence biological outcomes. In much of this work, sex and gender are treated as distinct explanatory variables: sex is said to index “biological” factors, whereas gender indexes “social” or “experiential” ones. The distinction, which is based on 20th -century conceptualizations of “nature” and “nurture” [1, 2], is useful in that it reminds us that not all variation can be explained by genes or hormones [3]. At the same time, such frameworks rest on an implicit partitioning of “biology” and “environment” into separable domains, such that their contributions are conceptualized—and analyzed—as independent or additive sources of variation. In biomedicine, keeping these sources of variation conceptually separated is not only common but formally institutionalized [4–10].

The relative weight given to either set of factors varies across fields and research traditions. Sex differences in the biomedical sciences are often attributed largely to physiological factors such as genes or hormones (discussed in [11]); in evolutionary psychology, to natural or sexual selection [12–14]. Developmental accounts of sex and gender have long incorporated multiple sources of influence [15], including hormone–experience interactions (e.g., [16–20]). Many of these approaches move beyond strictly biological determinism by recognizing that gendered outcomes emerge from multiple interacting influences. At the same time, they typically retain a conceptual distinction between biological and social factors, treating them as separable components whose effects combine over the course of development.

Approaches that silo biological and experiential processes can overlook how they are mutually constitutive over development [21–25]. When sex and gender are treated as separable causes, the developmental processes through which biological and social factors become intertwined are difficult to represent. To acknowledge this “socio-biological intertwinement” [26] (p. 50), some authors have introduced terms such as “sex/gender” [26] or “gender/sex” [27, 28]. The uptake of such terms has, however, been mixed.

The difficulty of separating the effects of sex and gender is intrinsic to the concept of “sex/gender entanglement,” a term used by Springer et al. [29] to express metaphorically that sex differences in human health always reflect the contribution of both sex and gender. The authors borrowed the term from quantum mechanics, where elements (e.g., particles) are characterized as “inextricably interwoven factors” (p. 1818). According to DuBois et al. [30], entanglement metaphorizes not only the inseparability of sex and gender but also their co-constitution. As an example, the authors mention testosterone; typically considered a sex-related factor, the hormone is sensitive to gendered contexts, behaviors, and experiences. Similarly, Ritz et al. [31] saw sex and gender “as co-constituted and in dynamic, looping relationships, with every factor understood as being both sex-and-gender at the same time …” (p. 99). Saldanha et al. [32] defined gender/sex entanglement as a “… reiterative interaction … between sex and gender over development” (p. 28). Whether these definitions reflect similar or distinct conceptualizations of entanglement, the adoption of the term itself does not simply suggest caution in interpreting statistical differences between groups. Instead, it explicitly draws attention to the dynamic relationship between sex and gender, one that cannot be captured by additive models (i.e., “sex and gender”).

As an increasing number of scholars adopt frameworks that operationalize sex/gender outside of those assumptions, updates are required to biological theories, methods, and concepts consistent with these ambitions. What is needed is a framework that formalizes this entanglement by modeling development as an ongoing interaction among processes that are not readily decomposed into independent “biological” and “environmental” components [33]. Here, we briefly review the ideas foundational to developmental and dynamic systems theories, which are theoretical frameworks that provide both conceptual and methodological tools to investigate systems in which complex patterns (including behavioral patterns) emerge from the dynamic interplay of multiple factors over time. These features make them well-suited for questions about sex and gender; however, with a few notable exceptions to be discussed below, they remain largely absent from the sex/gender literature.

Biomedical and biological researchers collecting and analyzing sex-disaggregated data are increasingly aware of the concept of sex/gender entanglement, and there has been much interest in developing or co-opting tools that can incorporate the concept into research designs and data interpretation [30, 31, 33]. Here, we provide a brief overview of developmental and dynamic systems theories for readers interested in and newly acquainted with the concept of sex and gender from an entanglement point of view. In the first section, we provide some background on developmental and dynamic systems approaches, some of their tenets, and seminal research in developmental psychobiology and psychology. Next, we discuss examples of how these approaches have informed sex/gender research in psychology. These examples have the potential to inform the process of biological research, for example by modifying the way we design studies, interpret results, and communicate findings.

## Developmental and dynamic systems frameworks: An overview

Developmental and dynamic systems theories (DST and DyST, respectively [34]) are related but distinct theoretical perspectives emerging from a variety of sources [34–42]. These theories, which share some key tenets, have been productively adopted in the study of behavior (e.g., [42–46]). We discuss each in turn below.

### Developmental systems theory (DST): Interrogating development beyond the nature-nurture divide

While the term “developmental system” was first coined by Waddington [47], DST became established in the 1990s [34] in the fields of philosophy of science (e.g., [39, 40]) and developmental psychobiology (e.g., [48]). Its intellectual roots can be found in the work of scholars such as Kuo, Schneirla, and Lehrman, who viewed development as a step-by-step construction shaped by multiple factors interacting with each other in complex ways (e.g., [35, 49, 50]) instead of a linear process of unfolding based on preexisting instructions or blueprints (e.g., [51]). The latter views have often reduced explanations of the origins of behavior to two possible “causes” or sets of processes: as due to information *inside* the organism (typically the genes, like in the case of instinctive behavior according to Lorenz [51]) or information *outside* it (e.g., the environment). This distinction, based on the “nature versus nurture” problem formulated by Galton [52] and associated dichotomies (e.g., innate-learned, biological-cultural), has been challenged on several grounds. First, this dichotomy is unable to capture the complexity of developmental processes. For example, genes and their products are involved in learning, and learning is at the core of many behaviors considered “instinctive,” such as hunting [53]. Second, deprivation experiments used to identify instinctive or “innate” (vs. learned or acquired) behaviors cannot methodologically eliminate all potential experiences; the laboratory and the organism itself are environments [54]. Third, assuming that information about the state of an organism exists somewhere before its existence, also known as preformationism [39, 40], is inherently illogical. Finally, there has been confusion concerning the meaning of “nature” and “nurture” and the type of questions surrounding how behaviors and other traits come to be [55]. While repetitively rejected as obsolete, the nature-nurture distinction and related dichotomies can still be found in current scientific discourse [56], particularly regarding questions of sex and gender [1, 2, 57–59]. It is this dichotomization that the concept of sex/gender entanglement aims to avoid.

One of the key perspectives of systems frameworks is to view living organisms as open complex systems: they maintain themselves by communicating with their surrounding environments through continuous exchanges of energy and matter [60]. These exchanges are necessary for the growth, increasingly complex organization, and behavior of organisms; new structures and functions emerge during development through a process of self-organization, rather than as the result of a pre-existing set of rules. Traits result from the interaction of multiple factors distributed within and outside of the organism; thus, organisms cannot be considered separately from their environments (physical, chemical, social, cultural, historical, etc.). The unit of study, therefore, is not the organism per se but rather the “developmental system”—the organism *and* its environments [61].

Oyama [61] described the relation between organism and environment as follows:

The organism-environment relations in a developmental system are indissoluble, not in the sense of being unalterable or more fundamental than other relations—indeed, both relations and constituents are changing all the time, and many developmentally and evolutionary phenomena depend on the organisms selecting and changing their environments. The ties are indissoluble in the following sense: that no organism can exist or even be characterized independently from a richly elaborated world on many scales of magnitude, that causal responsibility for the whole or a for a trait cannot be partitioned among the parts of the system, and everything the organism does and is rises out of this interactive complex, even as it affects that very complex. (pp. 168–169)

Oyama [62] described the organism and environment as continuously constructing each other through “constructive interactionism” (p. 184). Unlike more traditional usages of “interaction,” such as in statistics where factors operationalized as independently varying from one another interact to predict developmental outcomes, interactions in DST are assumed to be open-ended, that is, their directions and outcomes evolve and change over time and across different organism-environment relationships. Many classic examples from developmental science illustrate the importance of timing, often conceptualized as “critical,” “sensitive,” or “organizational” periods of development. For example, exposure to light and to language during particular periods of development are required for typical neural connectivity in visual cortex [63, 64] and development of language skills [65], respectively. The existence of sensitive periods for visual and language development might seem to suggest a maturational timetable unfolding from within. But from a DST perspective, the time windows themselves, and the degree of context-sensitivity within them, emerge from ongoing organism–environment interactions. According to DST, development is contingent on these interactions throughout life and on the very conditions in which the organism develops (e.g., [1, 66]). Thus, DST offers an alternative view of the critical period as not simply a window during which the environment can have effects on a passive nervous system but as an active, dynamic process during which each component informs, shapes, and constrains the other.

The organism’s contribution to its own development was exemplified in work by Gottlieb in a series of elegant experiments on the response of mallard ducklings to the maternal assembly call [48]. This call is used by the parent to ensure that its ducklings follow it out of the nest after hatching. Ducklings show a strong preference for the call at hatching, compared with calls from other species, even when eggs are incubated in isolation. Instead of assuming an “innate predisposition” for the call, Gottlieb tried to identify the developmental precursors of the preference by considering what ducklings experience under typical conditions. Gottlieb observed that the ducklings started vocalizing before they hatched and wondered whether the resulting auditory experience of hearing their own vocalizations shapes the ducklings’ preferences. Indeed, experimentally preventing the ducklings from vocalizing eliminated the preference for the parents’ calls. Further, when eggs were incubated in isolation and exposed to a chicken’s call during the pre-hatching period, devocalized ducklings preferred the chicken call. Thus, early preference could be established through a variety of routes, some in line with traditional expectations about developmental exposure to stimuli (hearing the maternal call) and others that were considered non-obvious at the time (the ducklings’ own vocalizations or those of their siblings before hatching). The expansion of the functional unit of an organism to include its environment extended Gottlieb’s attention to factors, such as experiences, contextual variables, and the organism’s own behavior, that might be important for a given developmental outcome.

There is by now a larger literature describing contributions to development from sources that were, historically, non-obvious (e.g., [67–71]). Moore [72], for example, while studying the development of sexual behavior in rats, made the unexpected discovery that both behavior and the underlying neural mechanisms were influenced by the behavior of the mother during the pups’ first weeks of life. At the time, adult male copulatory behaviors and the underlying circuitry were understood to be “organized” during an early sensitive period by exposure to endogenous testosterone [73, 74]. Moore showed that testosterone in the pups’ urine induced anogenital licking by the mother [75], which in turn itself contributed to the adult behavior [76], perhaps by stimulating the development of neural circuits involved in mounting and intromission [77]. Male pups were more likely than female pups to assume a supine position, stimulating maternal licking; male pups also released more urine after a licking bout, which stimulated even more licking [72]. This work, and the related work that followed it (e.g., [78, 79]) illustrated the rich web of reciprocal influences between pups and their mothers and reminded us that early social contingencies, which traditionally could be viewed in terms of organizational or critical periods, under DST create important opportunities for constructive interaction.

The examples of the ducklings and the rat pups illustrate a way of thinking about causality that lies at the heart of DST. Developmental systems are conceptualized as nested levels of organization; components at each level (e.g., genetic, cellular, neural, behavioral, environmental) influence each other dynamically across time [48, 80, 81]. Changes at one level of organization have repercussions at other levels; in turn, each level receives feedback from the levels it affects, in a never-ending loop of exchanges in which causes become effects and vice versa [45, 60, 62]. In the example above, rat pups and their mothers engage in a complex series of interactions that continuously shape the other’s behavior and physiology [72]. Similarly, in human development, the shift from crawling to walking provides toddlers with new points of view and affordances, which in turn shape their visual, motor, and cognitive development as well as their caregivers’ behavior (e.g., [82]). Although contributions of a variable can be isolated experimentally, the effect of that variable is contingent on a myriad of other factors that characterize the dynamic state of the system at that time. As a result, its contribution cannot be measured independently from those with which it interacts and on which it depends. As Oyama [62] wrote, “Starting an account with genetic transcriptions, and treating the DNA as an “independent variable” that “initiates” an interesting cascade of events, leads only too easily to obliterating from the causal landscape the events and conditions that brought that transcription about” (p. 182).

This “causal landscape” conveys a dynamic and distributed view of causality [62] and captures aspects of developmental change that cannot be uniquely studied, or even discovered, with typical controlled experiments. Although such experiments allow researchers to isolate the effect of specific variables, the precise role of those variables depends on factors, often considered uninteresting, that are kept constant and are thus excluded from the analyses [62]. As a consequence, their contribution to the phenomenon under investigation remains unexplored. But this contribution might be crucial. In a classic demonstration of variation from unexpected sources, differences in maze learning between “maze-bright” and “maze-dull” lines of rats disappeared when the rearing environment was manipulated to be either more deprived or more enriched than usual [83]. In humans, changing the objects in a classroom from those associated with a particular masculine stereotype (e.g., video games, computer parts, Star Trek posters) to non-stereotypical (water bottles, nature posters, coffee mugs) reversed a sex/gender difference in self-reported interest in computer science [84]. While these studies were not developed within the DST tradition, they exemplify how attention to the organism’s experiences can shed light on factors that might be important for the development of a behavior.

DST holds that development emerges from ongoing, reciprocal interactions among genes, organisms, and environments, with no single level (such as genes) having causal priority. It avoids metaphors such as pre-specified programs and blueprints, emphasizing instead that developmental outcomes are constructed over time through context-dependent processes across multiple scales, e.g., proteins, neuronal circuits, and social interactions. Some of these themes can also be found in dynamic systems approaches, to which we now turn.

### Dynamic systems theory (DyST): Traits as emerging and softly assembled

The DyST framework spans across disciplines and includes work on a diverse range of phenomena, including the formation of snowflakes and clouds, the patterns observed in chemical reactions, animal behavior, and developmental changes in living systems. Like DST, DyST focuses on change in complex systems that self-organize; that is, their organization emerges without a centralized control or a pre-existing program. Think of the complex moving patterns created by flocks of birds or schools of fish: their organized behavior relies on a few simple rules followed by each member interacting with their neighbors, not on directions orchestrated by a leader [85]. Unique patterns emerge across time, shaped by the interactions of elements contributing to the system. These interactions are often nonlinear: small changes can have large, small, or no consequences, depending on the constraints imposed by other elements at play and the history of the system. The organization that emerges differs from that of its constituent elements [42] and in turn affects their activity [46, 86]. Therefore, systems frameworks tend to reject reductionist views in that no level of analysis or constituent element is privileged.

Scholars working within a dynamic systems framework describe and explain how coordinated patterns emerge in complex systems [45]. Analyses of these phenomena are designed to capture the emergent (i.e., non-linear) properties of dynamic systems, which are described in terms of cascading processes and cross-scale interactions. Mathematical tools include, among others, nonlinear functions, attractors, and fractal analysis (e.g., [42, 45, 46]). These methods require tracking changes over time, collecting many data points (e.g., response times, heartbeats) and analyzing variability across timescales for each individual. When arranged in a time series, individual data typically look very noisy. This noise, however, can take on a predictability such that the patterns seen across large time scales (e.g., days) can look similar to those seen across smaller ones (e.g., minutes) -- this phenomenon, known as statistical self-similarity, is a feature of fractal structures and a key characteristic of most physiological processes [87]. While variability is often treated as noise, some of it actually reflects non-linear and irregular influences from other sources (e.g., autonomic, hormonal, behavioral in the case of heart rate). These influences occur at different timescales in different systems. The presence of fluctuations that correlate across different timescales is a marker of coordinated activity and interdependence in self-organizing systems. A description of these concepts as applied to the analysis of response times in cognitive tasks is provided by Van Orden et al. [46].

The DyST framework has been productively adopted in psychology. Applied to human development, DyST rejects the metaphor of the brain as a computer or machine [45, 88, 89]. Instead, DyST recognizes that complex systems are always in flux; stability, when present, is contingent and maintained, not fixed [90]. Scientists working within the tradition of ecological psychology have explored the interactions between perception and action and how these interactions contribute to cognition [44–46]. Recent applications of a DyST framework have been proposed in affective and behavioral neuroscience [91–94].

Some of the most influential applications of DyST to human development are found in Thelen’s empirical work on motor development in infants, which focused on walking and reaching [42, 95]. When Thelen started her research program, the dominant view of how infants learn to walk was that infants progress through a series of stages, from stepping reflexes to integrated walking, thanks to maturational changes in the central nervous system; these changes were considered by some to be genetically programmed (see [42]). Thelen challenged this explanation and demonstrated that behaviors exhibited by infants were shaped by a variety of factors and contextually situated. Stepping, for example, is typically present in the first two months of life, then disappears, and finally reappears between 8 and 10 months. This progression was initially interpreted as evidence of progressive central control: reflex-like at first, the stepping behavior would then be suppressed by higher neural centers to be performed intentionally. Thelen showed that the behavior is in fact flexibly expressed in contexts such as high arousal, water immersion, and when the infant was on a treadmill. Infants on the treadmill adopted a flexible stepping rhythm, in synchrony with the treadmill speed. Even on a split-belt treadmill in which the speed of the two belts differed, infants could modify their step lengths independently for each leg [42]. Stepping was absent or declined rapidly with weight gain or when weights were applied around the infants’ thighs. These remarkable adaptations illustrate that, even in infants before they can walk on their own, coordinated movement patterns emerge through active, ongoing calibration between the organism and its environment, rather than from an internally fixed motor program.

According to Thelen and Smith [42], the demonstrated complexity of these patterns defies our propensity to identify one internal cause as responsible for the development of stepping behavior, for example the “maturation” of the motor cortex. Whether the infant expresses a behavior depends on the context, which needs to be part of the explanation for that behavior. Behavioral solutions emerge, “soft-assembled,” that is, flexibly and based on components that are present and available at each level—physiological, social, etc., and at precise moments in time [90]. The resulting temporary outcomes are not predetermined; a system’s end-state “is not coded anywhere” (42; p. 49) and “Order is not ‘in there,’ but is created in the process of the action”(p. 63). Likewise, there are no privileged causes. The sophisticated locomotor behavior of pre-walking infants on a treadmill cannot be explained without including the treadmill as one of the causes of that behavior, along with other necessary factors, such as neural pathways and muscles, at the relevant stage of development [42].

The contingency and soft-assembly of behavioral patterns, in addition to the contribution of the organism’s own behavior, are evident also in the range of solutions that infants create when learning to reach for objects. Thelen [90] listed a number of requirements for this seemingly simple task. Besides the motivation to reach for an object, which depends on previous interactions with the environment, an infant needs a visual system with the capacity to fixate on objects and gauge their location in space; the ability to plan actions and make corrections based on visual feedback; the ability to stabilize their body while extending their arms and fine-tune their movement; and the ability to remember previous actions and their outcomes. All these components, which are also needed for other tasks, are necessary for learning to reach for objects. Different solutions emerge in different infants depending on some initial conditions, for example body size and energy levels, which constrains how their bodies move, and on how these components are engaged and coordinated. Cognition subsequently develops from bodily interactions with the world. From this perspective, cognition is “embodied,” meaning that cognitive processes emerge from bodily experiences [42].

Like DST, DyST assumes that biobehavioral phenomena result from dynamic change and conceptualizes causality as distributed and contingent on organism–environment interactions. DyST adds more formal focus on real-time dynamics, state spaces, and pattern formation to capture the dynamic nature of how living systems behave and develop. Both frameworks emphasize the emergence of developmental outcomes from ongoing, context-dependent interactions; rather than treating behavioral variability as biologically or socially determined, these approaches highlight how it can arise, shift, and be maintained through ongoing interactions across physiological, behavioral, and environmental levels. Such approaches provide key concepts and methods to interrogate sex and gender as entangled. Below, we explore research adopting these frameworks to the study of sex/gender.

## DST/DyST in sex/gender research

Because DST and DyST move beyond nature–nurture frameworks by treating development as an emergent process, they are especially useful for examining complex, multifaceted phenomena such as sex/gender [27, 33]. Despite the many potential benefits of using DST/DyST to interrogate the development of sex/gender-related variation, we are aware of only a few research programs that have done so. In this section, we highlight work from three independent research groups that have taken a DyST approach to understand gender-related phenomena as softly assembled, contextually situated, and having distributed, complex causality. Martin and colleagues [96, 97] and Diamond and colleagues [98–100] have used DyST frameworks to explore the development of gendered behavior and gender identity; Fausto-Sterling and colleagues have applied a DyST conceptual approach to sex/gender entanglement, interrogating the development of sex/gender differences in early childhood [101–104]. The examples within each program adopt a DyST framework but differ in focus, allowing us to illustrate flexible application of DyST to distinct but related problems.

### DST/DyST and the development of gendered behavior

Work by Martin and colleagues illustrates how DyST can be used to understand the relevance of the structure of social spaces in the emergence of gendered behavior. In DyST, predictable and flexible developmental trajectories are driven by “attractors,” which represent preferred states (e.g., focused attention) or activities (e.g., reading) that are likely to occur even in the face of minor perturbations [105, 106]. Attractors can be identified empirically by converging evidence—frequent visits to a region, rapid first entry, and short return times. If a perturbation causes a deviation from the attractor, the individual is likely to return eventually to that state or activity. Martin et al. [96] utilized the concept of attractors as an analytical tool to understand how sex segregation in young children’s peer groups emerges and stabilizes over time. They conceptualized preschool classrooms as self-organizing social systems in which dynamic, moment-to-moment interactions develop into stable patterns. Using state space grid analysis, the authors mapped children’s interactions across dimensions such as peer sex and behavioral style, treating each change in behavior as a point in the state space and children’s social histories as trajectories through that space. This method revealed the geometry of the social system – where children went, how often they returned, and which interactional states exerted the strongest pull. The results showed that same-sex play functioned as a strong attractor early in the preschool years. The authors concluded that robust sex segregation can arise from relatively small initial tendencies, such as expectations that same-sex peers will share interests, which are then reinforced through repeated interactions. By tracking how these attractor landscapes stabilize and strengthen over time, the authors showed how sex segregation is not simply chosen or imposed, but instead self-organizes during everyday social interaction.

In later work, Martin and colleagues shifted their focus from interactional space to developmental time. Notably, the DyST approach does not assume or require that children’s preferences for same-sex peers remain static. Instead, it allows for frequent departures from sex-segregated play while still producing a predictable pattern across time. Martin and colleagues [97, 107, 108] argued that understanding sex segregation requires attention to *patterns* of change across moments, contexts, and timescales, not just to average levels of gender-typed behavior. By examining sex-segregated play behavior on a day-to-day timescale, they illustrated how stable developmental regularities can arise from fluctuating behavior: children may alternate between same- and other-sex play across situations, yet still show reliable tendencies to return to same-sex peers over time. The fluctuations in behavior occur on timescales that are highly variable and not correlated with past behavior, but assume a fractal structure: the short-term variability seen over days is nested inside long-term variability, measured over weeks or months. Anticipating and leveraging this nesting revealed that short-term influences, such as the attractiveness of a particular play activity, interact with long term influences, such as a friendship, to shape gendered behavior [107]. From this perspective, variability in sex-segregated behavior is not a nuisance, but rather a key source of evidence about the strength and flexibility of gendered social organization.

By showing how behavioral patterns such as sex segregation emerge from day-to-day interactions rather than fixed traits, Martin and colleagues identified small, early shifts in social context that can alter developmental trajectories. These DyST-informed analyses help us understand how social environments shape exposure, opportunity, and experience—processes that are ultimately important for mental health, learning, and social development.

### DST/DyST and sexual fluidity

In humans, female sexuality is often conceptualized as more flexible, or fluid, than male sexuality, a characterization that could lead to misinterpretation of female behavior as simply random rather than changing in a developmentally meaningful way [98]. Because DyST regards change as constitutive of development rather than as noise, it can resolve apparent variability as patterned rather than random. Whereas Martin and colleagues used within-individual variability to reveal emergent structure over space and time, Diamond’s work in this area has treated variability over time as the object of study, using dense longitudinal data to show how identities and attractions reorganize within individuals across days, years, and developmental periods. In an application of DyST to understand the development of same-sex sexuality, Diamond [98] interviewed approximately 80 women (identifying as lesbian, bisexual, or undefined) every 2–3 years over a decade. During the course of the study, two-thirds of the women changed their identity, some more than once. Changes in identity can be nonlinear and abrupt, prompted by an event such as a kiss, an Oprah episode, or a course in Women’s Studies [98]. Abrupt shifts, which in traditional approaches would be relegated to a wastebasket category of exceptions or inconsistencies, can be accommodated by approaches using DyST because it treats dynamism and variability as a fundamental feature of any system.

DyST can be applied to shorter timescales. Using a DyST approach, Farr et al. [100] found that while higher sexual attraction and more sexual activity were reported in lesbian women compared with bisexual and “fluid” women, day-to-day oscillations in same-sex attraction were similar in magnitude across groups. Their finding stood in contrast with the belief that lesbian women are more “stable” in their attractions than are bisexual women. Rather, the study by Farr et al. supported the idea that some degree of plasticity may be common to female same-sex sexuality. These fluctuations, which could be a fundamental characteristic of any identity, would not be detected using other types of research designs.

Traditional “coming out” or “organismic” models of sexuality, which assume rigid stability in mature sexual identity and behaviors, can fail to capture their dynamic nature. In a study of same- and other-sex attraction in men and women, Diamond et al. [99] found that attractions in women (degree of attraction to same- or other-sex) were less stable, day-to-day, than men’s. When asked to recall the development of sexual attractions in post-adolescence, however, men and women recalled similar changes. Moreover, the extent of recalled change did not predict the stability of adult attractions. Diamond et al. concluded that sexual identity does not appear to be uniformly more fluid in women than men. Instead, gender differences in the capacity for change can depend on the timescale assessed. The approach used in such studies, focusing on multiple measures and dense within-individual sampling, allowed the simultaneous assessment of both stability and change over time. In the decade-long study conducted by Diamond [98] described above, for example, although women’s own self-labeling (e.g., lesbian or bisexual) changed dramatically over time, their self-reported attractions (e.g., to women vs. men) remained remarkably stable. Dynamical systems models are ideally suited to modeling global stability combined with local variability [100]—a hallmark of dynamic systems.

The work of Diamond and colleagues has demonstrated that DyST can alter our methods and analysis but requires a type of data collection we may not be used to—longitudinal observation over both short and long stretches of time. The focus is on within-individual variability, as opposed to identifying factors that differ between individuals in two categories. The trick is that any particular factor might have a different effect, depending on where the individual is in their development. In the context of sexual identity, a person with similar degrees of attraction to men and women might be nudged in a particular direction by one relationship, but not so for someone with attractions leaning more strongly in one direction. Thus, Diamond recommends charting the most influential variables—or attractors—over time and testing hypotheses under conditions that include the relevant ranges of variability. As explained by Farr et al. [100], the conditions influencing propensities for change can both propel and constrain development, channeling individuals into predictable yet flexible trajectories. The task at hand is to identify and interrogate the conditions giving rise to those trajectories and the boundaries between them [99].

### DST/DyST and the development of gender/sex differences in early childhood

The research programs led by Martin [96, 97, 108] and Diamond [98–100] demonstrate how a DyST framework can bring unique and valuable perspective to our understanding of gender and gendered behavior as self-organized, dynamic, and multiply determined. Fausto-Sterling and colleagues have substantially expanded the potential impact of DyST by considering how it might be applied across multiple levels of biological and social organization to more directly engage with the problem of gender/sex^1^ entanglement. In the first of a two-part set of papers entitled “Sexing the baby,” Fausto-Sterling et al. [103] reviewed evidence of gender/sex differences in early childhood with the goal of identifying targets for DyST-based investigations. For example, they identified many unaddressed questions regarding gender/sex variation in endocrine development: how do dynamic interrelations between the mother and infant (both prenatally and postnatally) contribute to gender/sex-variation in the infant endocrine system? What are the metabolic characteristics and the sites of action of hormones during early development? What roles do hormones such as testosterone play in infant bodily functions and development? What is the role of bodily experience in variation in neonatal hormones levels? In raising these questions, the group called attention to the types of variables (e.g., bodily experience, parent-infant interaction) and research questions (e.g., the role of early experiences in gender/sex variations in hormones) that could be studied using a DyST approach to interrogate the development of gender/sex and related identities.

In the second paper in this series [104], the authors proposed a model of development as socially and culturally shaped; variation in physiology and behavior emerges through differences in the way organisms recurrently experience their environment. Particularly important is the social environment, through which values and beliefs, including those about gender/sex, become integrated into development. For instance, early dyadic interactions between parents and infants, if variable by the gender/sex of the infant on average, could contribute to gender/sex variation in brain weight, cortical maturity, and sensory development. These phenotypes could, in turn, affect communication between infants and caregivers. Thus, within this model, bodily and behavioral variations between boys and girls can be considered to both inform and be shaped through constant interactions between the organism and its environment. Each bodily or behavioral trait can be considered only temporary, existing at particular moments in time (“softly assembled”) and influencing future development in nonlinear ways. For example, differences in activity levels across gender/sex display an interesting temporal variability. During the first months of life, differences between boys and girls are relatively small (boys more active than girls, 80% overlap), even disappearing around four months of age. They then reemerge to be larger by the end of the first year (reviewed in 103). Understood within a DyST framework, this nonlinear pattern of differences in activity level indicates that the development of motor skills during this period is unstable and prone to destabilization by other factors, such as parental interactions. Here, DyST treats infant behavior and parental response as dynamically coupled rather than as independent sources of influence [103, 104]. Initially small differences between neonatal boys and girls progressively grow and stabilize as social interactions also increasingly differentiate.

The identification of gender/sex differences during precise periods of development allows further investigation into their potential repercussions at later periods. In addition, this type of finding sets the stage for elucidating bidirectional relationships between parental behavior and child development. In a study of gender/sex-related variation in mother-infant interaction styles, Fausto-Sterling et al. [109] analyzed data from 30 mother-infant dyads (15 mother-son and 15 mother-daughter) filmed monthly in their home environments, from three months to 12 months of age. The longitudinal design of the study made it possible to see changes over time—for example, the frequency with which mothers touched their infants decreased between three and 12 months. There were, however, gender/sex differences in the rate of decrease; mothers were touching sons more often than daughters between three and six months of age, but the gender/sex difference disappeared at older ages as touching decreased overall. The early gender/sex differences were particularly apparent with certain types of ecologically relevant interactions, a discovery made possible by the study’s naturalistic context. For example, early in development, whereas mothers engaged in more caretaking (wiping nose, diapering, feeding, etc.) with daughters, they engaged in more affectionate touch (tickling, nuzzling, kissing, etc.) with sons. Similarly, mothers lifted and jiggled sons more often than daughters. Whereas sons were more likely to be assisted by the mother herself when trying to sit up, girls were more likely to be propped in a high chair or other non-human assistive device [109].

Using the same mother-infant behavioral data, Eason et al. [101] employed a vector autoregression-based method to show that rates of maternal touching could be predicted by the infants’ own behavior. In an analysis of sons, for example, the ability to independently stand predicted increases in maternal behaviors such as rocking, lifting, and jiggling. In a separate analysis of daughters, crawling behavior decreased the same maternal behaviors. Although the exploratory analytical approach did not test directly whether the predictive value of standing or crawling differed significantly between boys and girls, the preliminary findings provide a foundation on which to base future comparisons across gender/sex. Such comparisons are critical because, as shown in this study, considering behaviors independently of their dynamic pairing with the environment can mask potentially relevant variation.

Taken together, these three research programs illustrate not only the utility of DyST, but why conventional categorical approaches are a poor fit for understanding sex/gender development. DST/DyST allows a variety of potential explanatory and moderating variables to be tracked across a range of naturalistic contexts and can accommodate and reconcile both change and stability, advantages that are critical when interrogating processes of sex/gender differences. The studies outlined here illustrate how sex/gender work grounded in DST/DyST can produce models, theories, and empirical evidence aligned with the conceptual ambitions of sex/gender entanglement.

## Implications of systems frameworks for sex/gender research

DST/DyST frameworks have been instrumental in moving fields such as developmental biology and psychology away from the nature vs. nurture divide and into a situated account of development. They now invite us to rethink the way we conceptualize sex/gender. Their adoption comes with several commitments with respect to how we conduct sex/gender research, the first being a shift in focus to development and process. Most explorations of sex/gender are focused on explaining group differences, or they treat the concept of entanglement as the contamination of “biological” sex by social and cultural influences. In contrast, a systems approach shifts the focus from finding correlates to describing how phenotypes dynamically emerge and change with time. Developmental studies, especially when conducted in naturalistic settings, provide a rich and detailed description of the organism’s experiences and expand the range of variables that potentially contribute to the emergence of sex/gender-related variation. Gottlieb’s work on ducklings’ sensitivity to the maternal call [48] and Thelen’s work on the development of locomotion [42], reviewed above, represent seminal work and classic examples of truly developmental approaches. Martin’s, Diamond’s, and Fausto-Sterling’s work, also reviewed above, exemplifies how such approaches can be adopted productively to both generate and address questions pertaining to sex/gender. Whatever the topic, systems theories stress that the focus of investigation is on the dynamic and constructive interactions between organisms and their environments (the whole developmental system). In this view, environments are not static providers of stimulation. They shape the development of the organism and *develop with* the organism and its activity; thus, organisms change their own environments in a reiterative and circular form of causality. Adopting research designs and methods that can document and describe these dynamic processes (e.g., [101]) is critical to understanding these relationships.

It is important to note that DST/DyST are not themselves statistical approaches – a multitude of different analytical approaches can be used within a DST/DyST framework. The chosen approaches need not be fancy; for example, Thelen et al. [110] used the analysis of variance in their experiments on infants’ stepping behavior; Gottlieb [111] used the Wilcoxon test to analyze the ducklings’ preferences. The more important considerations are, first, that if claims of sex differences are to be made, then the analytical approach must statistically compare the sexes. Too often, approaches informed by DST/DyST consist of complex models built within each sex, which does not allow direct sex/gender comparisons [112]. Second, whether sexes/genders are compared or not, separation of groups by sex/gender categories reifies binary conceptualization and can miss individual variation [2, 113]. These shortcomings of traditional approaches would ideally be taken into consideration when implementing DST/DyST frameworks.

Adopting a systems approach invites caution when we draw conclusions about causation without knowing the full developmental path of a given outcome. If organisms are constructed through reiterative, distributed, and circular interactions across their developmental systems, then, *a posteriori*, it becomes impossible to disentangle the distinct effects of “sex” and “gender,” however defined, on a given outcome. Our bodies are encultured; this enculturation is expected to look different across individuals. DST/DyST cautions against broad generalization about these processes, particularly when they are characterized in homogenous populations, environments, or cultures. This issue may be particularly salient in the field of developmental psychology, where over 50% of recent articles published in prominent journals failed to report any sociodemographic information (reviewed in [114]). Empirically, research designs and analytical strategies that include naturalistic observation of dynamic organism-environment interactions minimize potential bias introduced in more constrained testing environments and measurements. Such approaches, which are themselves biologically sound, avoid assigning variation only to “biological” causes; effects are contingent on the status of the organism at any given time. Because behaviors (including attitudes, attributions, identities) emerge based on the ongoing interaction of multiple elements, sex/gender differences cannot be described as “essential” (e.g., [115]) or “fundamental” (e.g., [116, 117]). They are all contingent on the dynamics of the system and the interactions at play.

Finally, a systems viewpoint precludes describing sex/gender-related traits as “preprogrammed” (e.g., [118, 119]) because, in this framework, there is no program or blueprint. All organisms and their behaviors develop through a process of construction. Some outcomes are more typical or less variable than others, but the processes that support those outcomes need not differ from those supporting less typical or more variable outcomes, as assumed by dichotomous views of development (e.g., “innate vs. learned” behaviors; [39, 40, 120, 121]). All phenotypes are the result of developmental processes. What distinguishes more stable vs. more variable outcomes is whether the resources that fuel development, distributed across the entire developmental system and common to both outcomes, are “reliably reconstituted” across generations [121]. Because they are multiple and interacting dynamically, variability is an intrinsic feature of any evolving living system.

DST and DyST have much to offer to the study of sex, gender, and their dynamic relationship. They can capture the complexity and emergent properties of living organisms in dynamic and constructive interactions with their environments. By rejecting preformationist ideas about how traits come to be and predetermined endpoints, these approaches move past obsolete dichotomous views of development (e.g., “nature vs nurture”) and recenter the focus on the conditions and processes associated with the emergence of complex forms and behaviors. As we have seen, some of the causes of change are non-obvious at first. Regardless, what emerges is always contingent on the *dynamics* among the elements at play. By adopting frameworks that allow us to describe how complex systems work, we can capture the richness of behaviors, attitudes, preferences, and attributions that characterize sex/gender entanglement and sex/gender-related variation.

## Data Availability

Not applicable.

## Footnotes

1 Throughout this section, we use the term “gender/sex” to be consistent with the authors’ usage.

## References

[1] Fausto-Sterling A. Sexing the Body: Gender Politics and the Construction of Sexuality. Basic Books; 2000.

[2] Maney DL, Duchesne A, Grossi G. Sex/gender entanglement: a problem of knots and buckets. Biol Sex Differ. 2025;16(1):85. 41168807 10.1186/s13293-025-00758-9 PMC12577278

[3] Ritz SA, Greaves L. We need more-nuanced approaches to exploring sex and gender in research. Nature. 2024;629(8010):34–6. 38693410 10.1038/d41586-024-01204-3

[4] IGH Learning – CIHR. https://cihr-irsc.gc.ca/e/49347.html

[5] Sex and gender in research policy | NIHR. https://www.nihr.ac.uk/about-us/who-we-are/policies-and-guidelines/sex-and-gender-research

[6] What is gender?. What is sex? - CIHR. https://cihr-irsc.gc.ca/e/48642.html

[7] Clayton JA, Tannenbaum C. Reporting sex, gender, or both in clinical research? JAMA. 2016;316(18):1863–4. 27802482 10.1001/jama.2016.16405

[8] Heidari S, Babor TF, De Castro P, Tort S, Curno M. Sex and gender equity in research: rationale for the SAGER guidelines and recommended use. Res Integr Peer Rev. 2016;1:2. 29451543 10.1186/s41073-016-0007-6 PMC5793986

[9] Kaufman MR, Eschliman EL, Karver TS. Differentiating sex and gender in health research to achieve gender equity. Bull World Health Organ. 2023;101(10):666. 37772198 10.2471/BLT.22.289310 PMC10523819

[10] Gompers A, Olivier MT, Maney DL. Training in the implementation of sex and gender research policies: an evaluation of publicly available online courses. Biol Sex Differ. 2024;15(1):32. 38570790 10.1186/s13293-024-00610-6 PMC10988906

[11] Williams JS, Fattori MR, Honeyborne IR, Ritz SA. Considering hormones as sex-and gender-related factors in biomedical research: challenging false dichotomies and embracing complexity. Horm Behav. 2023;156:105442. 37913648 10.1016/j.yhbeh.2023.105442

[12] Archer J. The reality and evolutionary significance of human psychological sex differences. Biol Rev. 2019;94(4):1381–415. 30892813 10.1111/brv.12507

[13] Ellis L. Identifying and explaining apparent universal sex differences in cognition and behavior. Personal Individ Differ. 2011;51(5):552–61.

[14] Geary DC, Bjorklund DF. Evolutionary developmental psychology. Child Dev. 2000;71(1):57–65. 10836558 10.1111/1467-8624.00118

[15] Blakemore JEO, Berenbaum SA, Liben LS. Gender development. New York, NY, US: Psychology Press; 2009.

[16] Berenbaum SA, Blakemore JEO, Beltz AM. A role for biology in gender-related behavior. Sex Roles. 2011;64(11):804–25.

[17] Berenbaum SA, Beltz AM, Bryk K, McHale S. Gendered peer involvement in girls with congenital adrenal hyperplasia: effects of prenatal androgens, gendered activities, and gender cognitions. Arch Sex Behav. 2018;47(4):915–29. 29318470 10.1007/s10508-017-1112-4 PMC9173056

[18] McHale SM, Kim JY, Dotterer AM, Crouter AC, Booth A. The development of gendered interests and personality qualities from middle childhood through adolescence: a biosocial analysis. Child Dev. 2009;80(2):482–95. 19467005 10.1111/j.1467-8624.2009.01273.x PMC3242364

[19] Sisk CL, Zehr JL. Pubertal hormones organize the adolescent brain and behavior. Front Neuroendocrinol. 2005;26(3–4):163–74. 16309736 10.1016/j.yfrne.2005.10.003

[20] Wallen K. Prenatal steroid hormones and sex differences in juvenile rhesus macaque behavior. In: VanderLaan DP, Wong WI, editors. Gender and sexuality development: contemporary theory and research. Cham: Springer International Publishing; 2022. p. 39–72.

[21] Bleier R. Science and Gender: A Critique of Biology and its Theories on Women. New York: Pergamon Press; 1984.

[22] Eagly AH, Wood W. The nature–nurture debates: 25 years of challenges in understanding the psychology of gender. Perspect Psychol Sci. 2013;8(3):340–57. 26172976 10.1177/1745691613484767

[23] Fausto-Sterling A. Myths of gender: Biological theories about women and men. Basic Books; 2008.

[24] Jordan-Young RM. Hormones, context, and “brain gender”: A review of evidence from congenital adrenal hyperplasia. Soc Sci Med. 2012;74(11):1738–44. 21962724 10.1016/j.socscimed.2011.08.026

[25] Unger RK. Toward a redefinition of sex and gender. Am Psychol. 1979;34(11):1085–94.

[26] Kaiser A, Haller S, Schmitz S, Nitsch C. On sex/gender related similarities and differences in fMRI language research. Brain Res Rev. 2009;61(2):49–59. 19406148 10.1016/j.brainresrev.2009.03.005

[27] Fausto-Sterling A. Gender/sex, sexual orientation, and identity are in the body: How did they get there? J Sex Res. 2019;56(4–5):529–55. 30875248 10.1080/00224499.2019.1581883

[28] van Anders SM. Beyond sexual orientation: Integrating gender/sex and diverse sexualities via sexual configurations theory. Arch Sex Behav. 2015;44(5):1177–213. 25772652 10.1007/s10508-015-0490-8

[29] Springer KW, Stellman JM, Jordan-Young RM. Beyond a catalogue of differences: A theoretical frame and good practice guidelines for researching sex/gender in human health. Soc Sci Med. 2012;74(11):1817–24. 21724313 10.1016/j.socscimed.2011.05.033

[30] DuBois LZ, Kaiser Trujillo A, McCarthy MM, editors. Sex and Gender: Toward Transforming Scientific Practice. Springer Nature; 2025.

[31] Ritz SA, Bauer G, Christiansen DM, Duchesne A, Trujillo AK, Maney DL. Operationalization, measurement, and interpretation of sex/gender. In: DuBois LZ, Kaiser Trujillo A, McCarthy MM, editors. Sex and Gender: Toward Transforming Scientific Practice. Cham: Springer Nature Switzerland; 2025. p. 87–112.

[32] Saldanha CJ, Bentley GR, Cornil CA, de Vries GJ, Dunsworth H, McCarthy MM. Entanglement of gender/sex dynamics in basic and developmental systems biology. In: DuBois LZ, Kaiser Trujillo A, McCarthy MM, editors. Sex and Gender: Toward Transforming Scientific Practice. Cham: Springer Nature Switzerland; 2025. p. 23–47.

[33] Ritz SA, DuBois LZ. Reframing the sex debate in scientific research: three frameworks to support rigor without rigidity. Biol Sex Differ. 2025;16(1):106. 41419956 10.1186/s13293-025-00808-2 PMC12717726

[34] Griffiths PE, Tabery J. Developmental Systems Theory: What does it explain, and how does it explain it? In: Lerner RM, Benson JB, editors. Advances in Child Development and Behavior. JAI; 2013. p. 65–94. 10.1016/b978-0-12-397947-6.00003-9 23834002

[35] Johnston T. Developmental systems theory. In: Blumberg MS, Freeman J, Robinson SR, editors. Oxford handbook of developmental behavioral neuroscience. Oxford University Press; 2010. pp. 12–29.

[36] Lerner RM, Greenberg G. The Heredity Hoax: Challenging Flawed Genetic Theories of Human Development. Taylor & Francis; 2025.

[37] Lewis MD. The promise of dynamic systems approaches for an integrated account of human development. Child Dev. 2000;71(1):36–43. 10836556 10.1111/1467-8624.00116

[38] Moore DS. The Dependent Gene: The Fallacy of “Nature vs. Nurture.” New York, NY: Holt Paperbacks; 2003.

[39] Oyama S. The Ontogeny of Information: Developmental Systems and Evolution. Duke University Press; 1985.

[40] Oyama S. The Ontogeny of Information: Developmental Systems and Evolution. Duke University Press; 2000.

[41] Oyama S, Griffiths PE, Gray RD, editors. Cycles of Contingency: Developmental Systems and Evolution. Cambridge, Mass.: MIT Press; 2001.

[42] Thelen E, Smith LB. A Dynamic Systems Approach to the Development of Cognition and Action. MIT Press; 1994.

[43] Franchak JM, Adolph KE. An update of the development of motor behavior. WIREs Cogn Sci. 2024;15(6):e1682. 10.1002/wcs.1682 PMC11534565 38831670

[44] Gibson J. The Ecological Approach to Visual Perception. Houghton Mifflin; 1979.

[45] Kelso JAS. Dynamic Patterns: The Self-organization of Brain and Behavior. MIT Press; 1995.

[46] Van Orden GC, Holden JG, Turvey MT. Self-organization of cognitive performance. J Exp Psychol Gen. 2003;132(3):331–50. 13678372 10.1037/0096-3445.132.3.331

[47] Waddington CH. Evolution of developmental systems. Nature. 1941;147(3717):108–10.

[48] Gottlieb G. Synthesizing Nature and Nurture: Prenatal Roots of Instinctive Behavior. Lawrence Erlbaum Associates; 1997.

[49] Miller DB. From nature to nurture, and back again. Dev Psychobiol. 2007;49(8):770–9. 18023009 10.1002/dev.20269

[50] Miller DB. The provenance and control of behavior: simplistic answers are doomed to fail. Ecol Psychol. 2009;21(2):131–7.

[51] Lorenz K. Über die Bildung des Instinktbegriffes. Naturwissenschaften. 1937;25(19):289–300.

[52] Galton F. English Men of Science: Their Nature and Nurture. London: Routledge; 1874.

[53] Bateson P, Mameli M. The innate and the acquired: useful clusters or a residual distinction from folk biology? Dev Psychobiol. 2007;49(8):818–31. 18023000 10.1002/dev.20277

[54] Lehrman DS. A critique of Konrad Lorenz’s theory of instinctive behavior. Q Rev Biol. 1953;28(4):337–63. 13121237 10.1086/399858

[55] Keller EF. The Mirage of a Space between Nature and Nurture. Duke University Press; 2010.

[56] Kohn GM. Revisiting T. C. Schneirla’s “Interrelationships of the ‘Innate’ and the ‘Acquired’ in Instinctive Behavior” (1956). Biol Theory. 2024;19(2):84–93.

[57] Duchesne A. Gender and sex entanglement in neuroscience. In: DuBois LZ, Kaiser Trujillo A, McCarthy MM, editors. Sex and Gender: Toward Transforming Scientific Practice. Cham: Springer Nature Switzerland; 2025. p. 113–38.

[58] Grossi G, Fine C. The role of fetal testosterone in the development of the Essential Difference between the sexes: Some essential issues. In: Bluhm R, Jacobson AJ, Maibom HL, editors. Neurofeminism: Issues at the Intersection of Feminist Theory and Cognitive Science. London: Palgrave Macmillan UK; 2012. pp. 73–104.

[59] van Anders SM. From testosterone to racialization to knobby knees: 15 years of gender/sex. Am J Hum Biol. 2025;37(6):e70090. 40545909 10.1002/ajhb.70090 PMC12183542

[60] von Bertalanffy L. General System Theory: Foundations, Development, Applications. New York: Braziller; 1968.

[61] Oyama S. The nurturing of natures. In: Grunwald, A, Gutman, M Neuman-Held EM, editors. On Human Nature. Springer Berlin Heidelberg; 2002. p. 163–70.

[62] Oyama S. 15 Terms in Tension: What do you do when all the good words are taken? In: Oyama S, Griffiths PE, Gray RD, editors. Cycles of Contingency: Developmental Systems and Evolution. MIT Press; 2001. pp. 177–93.

[63] Hubel DH, Wiesel TN. The period of susceptibility to the physiological effects of unilateral eye closure in kittens. J Physiol. 1970;206(2):419–36. 5498493 10.1113/jphysiol.1970.sp009022 PMC1348655

[64] Maurer D. Critical periods re-examined: evidence from children treated for dense cataracts. Cogn Dev. 2017;42:27–36.

[65] Mayberry RI, Lock E, Kazmi H. Linguistic ability and early language exposure. Nature. 2002;417(6884):38. 11986658 10.1038/417038a

[66] Marquardt AE, VanRyzin JW, Fuquen RW, McCarthy MM. Social play experience in juvenile rats is indispensable for appropriate socio-sexual behavior in adulthood in males but not females. Front Behav Neurosci. 2023;16:1076765. 10.3389/fnbeh.2022.1076765 PMC9899815 36755666

[67] Blumberg MS, Dooley JC, Sokoloff G. The developing brain revealed during sleep. Curr Opin Physiol. 2020;15:14–22. 32864534 10.1016/j.cophys.2019.11.002 PMC7450535

[68] Hampton NG, Bolhuis JJ, Horn G. Induction and development of a filial predisposition in the chick. Behav. 1995;132(5–6):451–77.

[69] Masataka N. Effects of experience with live insects on the development of fear of snakes in squirrel monkeys, *Saimiri sciureus*. Anim Behav. 1993;46(4):741–6.

[70] Miller DB, Hicinbothom G, Blaich CF. Alarm call responsivity of mallard ducklings: multiple pathways in behavioural development. Anim Behav. 1990;39(6):1207–12.

[71] Wallman J. A minimal visual restriction experiment: preventing chicks from seeing their feet affects later responses to mealworms. Dev Psychobiol. 1979;12(4):391–7. 456764 10.1002/dev.420120413

[72] Moore CL. The role of maternal stimulation in the development of sexual behavior and its neural basis. 1992; Ann N Y Acad Sci. 1992;662(1):160–77. 10.1111/j.1749-6632.1992.tb22859.x 1456637

[73] Breedlove SM, Jacobson CD, Gorski RA, Arnold AP. Masculinization of the female rat spinal cord following a single neonatal injection of testosterone propionate but not estradiol benzoate. Brain Res. 1982;237(1):173–81. 7074356 10.1016/0006-8993(82)90565-0

[74] Goy R. Experimental control of psychosexuality. Philos Trans R Soc Lond B Biol Sci. 1970;259(828):149–63. 4399060 10.1098/rstb.1970.0055

[75] Moore CL. Maternal behavior of rats is affected by hormonal condition of pups. J Comp Physiol Psychol. 1982;96(1):123–9. 7056895 10.1037/h0077866

[76] Moore CL. Maternal contributions to the development of masculine sexual behavior in laboratory rats. Dev Psychobiol. 1984;17(4):347–56. 6745497 10.1002/dev.420170403

[77] Moore CL, Dou H, Juraska JM. Maternal stimulation affects the number of motor neurons in a sexually dimorphic nucleus of the lumbar spinal cord. Brain Res. 1992;572(1):52–6. 1611538 10.1016/0006-8993(92)90449-j

[78] Champagne F, Diorio J, Sharma S, Meaney MJ. Naturally occurring variations in maternal behavior in the rat are associated with differences in estrogen-inducible central oxytocin receptors. Proc Natl Acad Sci U S A. 2001;98(22):12736–41. 11606726 10.1073/pnas.221224598 PMC60123

[79] Champagne FA. Epigenetic mechanisms and the transgenerational effects of maternal care. Front Neuroendocrinol. 2008;29(3):386–97. 18462782 10.1016/j.yfrne.2008.03.003 PMC2682215

[80] Gottlieb G. Probabilistic epigenesis. Dev Sci. 2007;10(1):1–11. 17181692 10.1111/j.1467-7687.2007.00556.x

[81] Johnston TD, Edwards L. Genes, interactions, and the development of behavior. Psychol Rev. 2002;109(1):26–34. 11863039 10.1037/0033-295x.109.1.26

[82] Adolph KE, Hoch JE. Motor development: Embodied, embedded, enculturated, and enabling. Annu Rev Psychol. 2019;70:141–64. 30256718 10.1146/annurev-psych-010418-102836 PMC6320716

[83] Cooper RM, Zubek JP. Effects of enriched and restricted early environments on the learning ability of bright and dull rats. Can J Psychol Rev Can Psychol. 1958;12(3):159–64. 10.1037/h0083747 13573245

[84] Cheryan S, Plaut VC, Davies PG, Steele CM. Ambient belonging: How stereotypical cues impact gender participation in computer science. J Pers Soc Psychol. 2009;97(6):1045–60. 19968418 10.1037/a0016239

[85] Couzin ID, Krause J. Self-organization and collective behavior in vertebrates. In: Slater PJB, Rosenblatt JS, Snowdon CT, Roper TJ, editors. Advances in the Study of Behavior. Academic Press; 2003. p. 1–75.

[86] Ladyman J, Lambert J, Wiesner K. What is a complex system? Eur J Philos Sci. 2013;3(1):33–67.

[87] Lipsitz LA. Physiological complexity, aging, and the path to frailty. Sci Aging Knowl Environ. 2004;2004:16. 10.1126/sageke.2004.16.pe16 15103055

[88] Ball P. How life works: a user’s guide to the new biology. Chicago, IL: University of Chicago Press; 2023.

[89] Nicholson DJ. Reconceptualizing the organism: From complex machine to flowing stream. In: Nicholson D, Dupré J, editors. Everything Flows: Towards a Processual Philosophy of Biology. Oxford University Press; 2018. p. 139–66.

[90] Thelen E. Dynamic Systems Theory and the complexity of change. Psychoanal Dialogues. 2005;15(2):255–83.

[91] Barrett LF. Context reconsidered: complex signal ensembles, relational meaning, and population thinking in psychological science. Am Psychol. 2022;77(8):894–920. 36409120 10.1037/amp0001054 PMC9683522

[92] Harrison LA, Gracias AJ, Friston KJ, Buckwalter JG. Resilience phenotypes derived from an active inference account of allostasis. Front Behav Neurosci. 2025;19:1524722. 10.3389/fnbeh.2025.1524722 PMC12098587 40416792

[93] Pessoa L, Medina L, Hof PR, Desfilis E. Neural architecture of the vertebrate brain: implications for the interaction between emotion and cognition. Neurosci Biobehav Rev. 2019;107:296–312. 31541638 10.1016/j.neubiorev.2019.09.021 PMC6996540

[94] Tretter F. “systems medicine” in the view of von Bertalanffy’s “organismic biology” and systems theory. Syst Res Behav Sci. 2019;36(3):346–62.

[95] Thelen E. Bernstein’s legacy for motor development: How infants learn to reach. In: Latash, ML ed. Progress in Motor Control. 1998;1:267 – 88.

[96] Martin CL, Fabes RA, Hanish LD, Hollenstein T. Social dynamics in the preschool. Dev Rev. 2005;25(3–4):299–327.

[97] Martin CL, Ruble DN. Patterns of gender development. Annu Rev Psychol. 2010;61:353–81. 19575615 10.1146/annurev.psych.093008.100511 PMC3747736

[98] Diamond LM. A Dynamical Systems approach to the development and expression of female same-sex sexuality. Perspect Psychol Sci. 2007;2(2):142–61. 26151957 10.1111/j.1745-6916.2007.00034.x

[99] Diamond LM, Dickenson JA, Blair KL. Stability of sexual attractions across different timescales: the roles of bisexuality and gender. Arch Sex Behav. 2017;46(1):193–204. 27873031 10.1007/s10508-016-0860-x

[100] Farr RH, Diamond LM, Boker SM. Female same-sex sexuality from a dynamical systems perspective: sexual desire, motivation, and behavior. Arch Sex Behav. 2014;43(8):1477–90. 25193132 10.1007/s10508-014-0378-z PMC4199863

[101] Eason EG, Carver NS, Kelty-Stephen DG, Fausto-Sterling A. Using vector autoregression modeling to reveal bidirectional relationships in gender/sex-related interactions in mother–infant dyads. Front Psychol. 2020;11:1507. 10.3389/fpsyg.2020.01507 PMC7419485 32848979

[102] Fausto-Sterling A. A dynamic systems framework for gender/sex development: from sensory input in infancy to subjective certainty in toddlerhood. Front Hum Neurosci. 2021;15:613789. 33897391 10.3389/fnhum.2021.613789 PMC8062721

[103] Fausto-Sterling A, Coll CG, Lamarre M. Sexing the baby: Part 1 – what do we really know about sex differentiation in the first three years of life? Soc Sci Med. 2012;74(11):1684–92. 21802808 10.1016/j.socscimed.2011.05.051

[104] Fausto-Sterling A, Coll CG, Lamarre M. Sexing the baby: Part 2 applying dynamic systems theory to the emergences of sex-related differences in infants and toddlers. Soc Sci Med. 2012;74(11):1693–702. 21862195 10.1016/j.socscimed.2011.06.027

[105] DiDonato MD, England D, Martin CL, Amazeen PG. Dynamical analyses for developmental science: a primer for intrigued scientists. Hum Dev. 2013;56(1):59–75.

[106] Thelen E, Smith LB. Dynamic Systems Theories. In: Damon W, Lerner RM, editors. Handbook of Child Psychology. 1st ed. Wiley; 2007. p. 258–312.

[107] DiDonato MD, Martin CL, Hessler EE, Amazeen PG, Hanish LD, Fabes RA. Gender consistency and flexibility: using dynamics to understand the relationship between gender and adjustment. Nonlinear Dyn Psychol Life Sci. 2012;16(2):159–84. 22452931

[108] Martin CL, Cook RE, Andrews NC. Reviving androgyny: a modern day perspective on flexibility of gender identity and behavior. Sex Roles. 2017;76(9):592–603.

[109] Fausto-Sterling A, Crews D, Sung J, García-Coll C, Seifer R. Multimodal sex-related differences in infant and in infant-directed maternal behaviors during months three through twelve of development. Dev Psychol. 2015;51(10):1351–66. 26372294 10.1037/dev0000033 PMC4580286

[110] Thelen E, Ulrich BD, Niles D. Bilateral coordination in human infants: stepping on a split-belt treadmill. J Exp Psychol Hum Percept Perform. 1987;13(3):405–10. 2958589 10.1037//0096-1523.13.3.405

[111] Gottlieb G. Experiential canalization of behavioral development: results. Dev Psychol. 1991;27(1):35–9.

[112] Rich-Edwards JW, Maney DL. Best practices to promote rigor and reproducibility in the era of sex-inclusive research. eLife. 2023;12:e90623. 37917121 10.7554/eLife.90623 PMC10622144

[113] Sanchis-Segura C, Forn C, Wilcox RR. From significant to meaningful: ATOMizing the study of sex differences and similarities. Front Neuroendocrinol. 2025;80:101228. 41371479 10.1016/j.yfrne.2025.101228

[114] Fausto-Sterling A. Rethinking gender/sex identity. Am J Hum Biol. 2025;37(4):e70044. 40235281 10.1002/ajhb.70044

[115] Baron-Cohen S. The essential difference. The truth about the male and female brain. New York: Basic Books; 2003.

[116] Cook KM, De Asis-Cruz J, Lopez C, Quistorff J, Kapse K, Andersen N, et al. Robust sex differences in functional brain connectivity are present in utero. Cereb Cortex. 2023;33(6):2441–54. 35641152 10.1093/cercor/bhac218 PMC10016060

[117] Ingalhalikar M, Smith A, Parker D, Satterthwaite TD, Elliott MA, Ruparel K, et al. Sex differences in the structural connectome of the human brain. Proc Natl Acad Sci. 2014;111(2):823–8. 24297904 10.1073/pnas.1316909110 PMC3896179

[118] Swaab D, Wolff SEC, Bao AM. Sexual differentiation of the human brain in relation to gender-identity, sexual orientation, and neuropsychiatric disorders. In: Pfaff DW, Volkow ND, Rubenstein JL, editors. Neuroscience in the 21st Century. Cham: Springer; 2022. p. 4347–78.

[119] van Honk J, Schutter DJ, Bos PA, Kruijt AW, Lentjes EG, Baron-Cohen S. Testosterone administration impairs cognitive empathy in women depending on second-to-fourth digit ratio. Proc Natl Acad Sci U S A. 2011;108(8):3448–52. 21300863 10.1073/pnas.1011891108 PMC3044405

[120] Blumberg M. Freaks of Nature: What Anomalies Tell Us about Development and Evolution. Oxford University Press; 2010.

[121] Moore DS, Lickliter R. Development as explanation: Understanding phenotypic stability and variability after the failure of genetic determinism. Prog Biophys Mol Biol. 2023;178:72–7. 36682588 10.1016/j.pbiomolbio.2023.01.003

